# Phenotypic Modulation of Cancer-Associated Antioxidant NQO1 Activity by Post-Translational Modifications and the Natural Diversity of the Human Genome

**DOI:** 10.3390/antiox12020379

**Published:** 2023-02-04

**Authors:** Angel L. Pey

**Affiliations:** Departamento de Química Física, Unidad de Excelencia de Química Aplicada a Biomedicina y Medioambiente e Instituto de Biotecnología, Facultad de Ciencias, Universidad de Granada, Av. Fuentenueva s/n, 18071 Granada, Spain; angelpey@ugr.es

**Keywords:** phosphorylation, acetylation, ubiquitination, intracellular degradation, ligand-dependent stability, cancer, neurological disorders

## Abstract

Human NAD(P)H:quinone oxidoreductase 1 (hNQO1) is a multifunctional and antioxidant stress protein whose expression is controlled by the Nrf2 signaling pathway. hNQO1 dysregulation is associated with cancer and neurological disorders. Recent works have shown that its activity is also modulated by different post-translational modifications (PTMs), such as phosphorylation, acetylation and ubiquitination, and these may synergize with naturally-occurring and inactivating polymorphisms and mutations. Herein, I describe recent advances in the study of the effect of PTMs and genetic variations on the structure and function of hNQO1 and their relationship with disease development in different genetic backgrounds, as well as the physiological roles of these modifications. I pay particular attention to the long-range allosteric effects exerted by PTMs and natural variation on the multiple functions of hNQO1.

## 1. Human NQO1: A Stress-Protein Associated with Disease

Human NAD(P)H:quinone oxidoreductase 1 (UniProt ID: P15559) is a soluble, typically cytosolic, dimeric protein at the hub of the antioxidant defense and stabilization of up to 50 different proteins, including p53 and HIF-1α [1,2]. Although hNQO1 has historically been labeled as a cytosolic enzyme, it is likely found in multiple subcellular locations [2,3]. As an enzyme, it catalyzes the two-electron reduction of a wide range of quinones to hydroquinones using NAD(P)H as a coenzyme (see Table A1 in [2]), displaying negative cooperativity regarding catalysis and FAD binding [4,5,6,7] and also detoxifying superoxide radicals [8]. Its many functions have been recently reviewed, and I refer to these excellent reviews for further information [1,8]. 

Naturally-occurring variants, as well as mimetics of post-translational modifications (PTMs) in NQO1, can lead to protein loss-of-function through different mechanisms [9,10,11,12,13]. In this review, I summarize current advancements in the study of NQO1 functionality depending upon post-translational modifications on its protein sequence, as well as by mutations and polymorphisms found in cancer cell lines and human populations with uncertain associations with disease (in contrast to the very common, largely inactivating and cancer-associated polymorphism P187S) [1,13,14,15]), which may highly determine NQO1 activity in different individuals. 

Recent advances in DNA sequencing technologies are revealing significant genetic variability among human populations [16]. About half of these mutations are of uncertain significance [17], found in only a single individual [18], and are missense mutations (vs. the human consensus genome) [18]. This genetic variability also causes variable responses to pharmacological treatments [19,20,21]. It is partially accessible from databases such as gnomAD (with information of over 140,000 human genomes/exomes; https://gnomad.broadinstitute.org/ accessed on 15 December 2022) and more disease-oriented ones such as ClinVar (https://www.ncbi.nlm.nih.gov/clinvar/ accessed on 15 December 2022) or dbGaP (https://www.ncbi.nlm.nih.gov/gap/ accessed on 15 December 2022). 

NQO1 is highly inducible upon stress through the Nrf2 or Ah pathways [1,8]. The Nrf2 regulatory pathway mediates the delicate balance between oxidative signaling and antioxidant defense, and it is likely that the NQO1 antioxidant properties and its modulation of reduced/oxidized forms of NAD^+^ play important roles [3,8,22,23,24,25]. The Nrf-2 pathway is associated with multiple human pathologies, including alcohol-induced liver disease, cigarette smoking, cancer and neurodegeneration [25]. The molecular details and physio-pathological implications of Nrf2 signaling have been extensively reviewed in the past [25]. As is essential for this manuscript, it must be noted that alterations in NQO1 activity are also associated with certain diseases linked, to different extents, with oxidative stress, such as cancer, Alzheimer’s disease, Parkinson’s disease and atherosclerosis [1,8]. Remarkably, certain genetic variations in NQO1 have been associated with cancer development, possibly due to a loss of activity and stability [1,26,27,28,29,30]. It is plausible that somatic or germline mutations may cause a predisposition to develop diseases such as cancer [30]. However, the roles of natural genetic diversity and post-translational modifications in NQO1, and their relationship with disease, are unclear. The main aim of this manuscript is to provide an update on these issues. 

## 2. NQO1: A Simple Dimer with Complex Behavior

**Figure 1:**
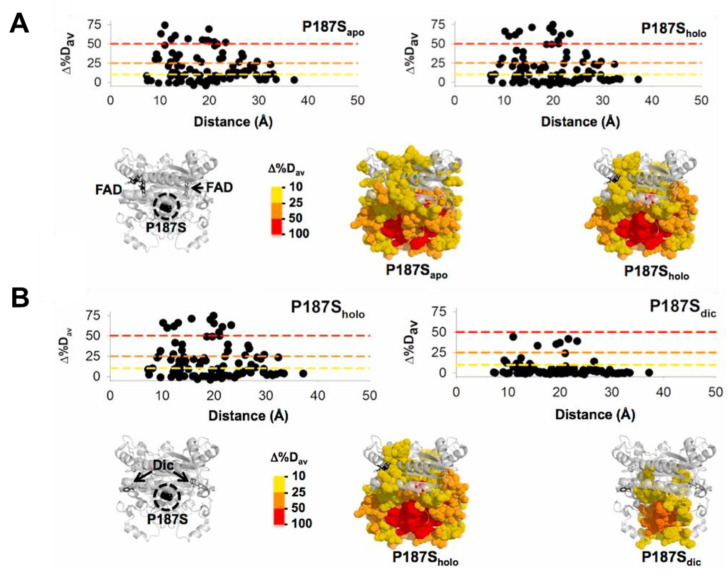
Long-range structural effects due to the cancer-associated P187S polymorphism in different ligation states (apo, no ligand bound; holo, with FAD bound; dic, with FAD and dicoumarol bound). Data are reproduced from [13]. The figure shows the effect of P187S (vs. the WT protein) in different ligation states (**A**, apo vs. holo; **B**, holo vs. Dic) considering over 100 protein segments by HDX (Δ%D_av_, a semiquantitative parameter calculated from the maximal differences in HDX; a positive value indicates a destabilizing effect) and regarding the distance to the mutated P187 residue. All figures were generated using the PDB code 2F1O [33].

Evolution has likely selected many mammalian proteins as oligomers to allow them to display complex regulatory behaviors. NQO1 seems to be an excellent example. Simply, as a dimer, NQO1 contains two active sites formed upon interaction of the large N-terminal domains (NTD), but requiring the small C-terminal domain (CTD) of the other subunit for efficient catalysis [28,31]. Both active sites, as well as the two domains, communicate in different functional ligation states along its catalytic cycle [4,9]. Detailed titration calorimetry experiments have revealed genuine negative cooperativity for FAD equilibrium binding to the apo-NQO1 protein [5]. In addition, binding of the competitive inhibitor dicoumarol (Dic) in calorimetric and steady-state activity assays also displays, in some instances, a degree of negative binding cooperativity [6,7]. Extensive functional and hydrogen–deuterium exchange (HDX) studies, as well as theoretical calculations, have supported the existence of long-range communication of local perturbations due to single amino acid changes and ligand binding to sites as distant as 30 Å [9,10,12,13,31,32] (Figure 1). This long-range conformational communication likely underlies the cooperative effects described upon ligand binding.

## 3. Post-Translational Modifications (PTMs) in hNQO1

**Figure 2:**
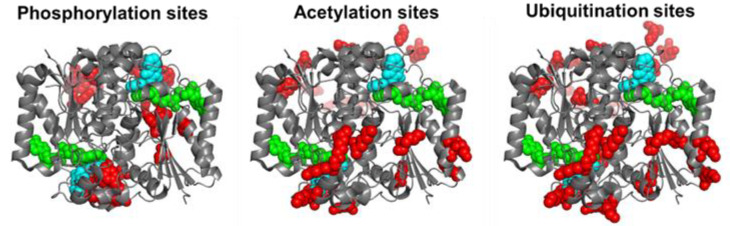
Structural location of phosphorylation, acetylation and ubiquitination sites of hNQO1 based on the data compiled in Phosphosite Plus^®^ [34]. Residues in red show those which are modified based on high-throughput analyses. I show the Dic molecules in cyan and the FAD in green. This figure was made based on the structure with PDB code 2F1O [33].

By 11 December 2022, the Phosphosite plus^®^ site (https://www.phosphosite.org/proteinAction.action?id=14721&showAllSites=true accessed on 11 December 2022) contained 12 phosphorylation sites (S13, Y20, S40, Y43, Y68, Y76, S82, Y127, T128, Y129, Y133 and S255), 9 acetylation sites (K31, K59, K61, K77, K90, K209, K210, K251 and K262) and 18 ubiquitination sites (K23, K31, K33, K54, K59, K61, K77, K90, K91, K135, K209, K210, K241, K248, K251, K262 and K271) for hNQO1. As can be seen, these sites are well-spread across the entire protein structure, and in the case of ubiquitination and acetylation, they often overlap (Figure 2). I must note that the functional consequences of only a few of these sites have been characterized in detail. These studies are described and discussed in this section.

### 3.1. Phosphorylation

**Figure 3:**
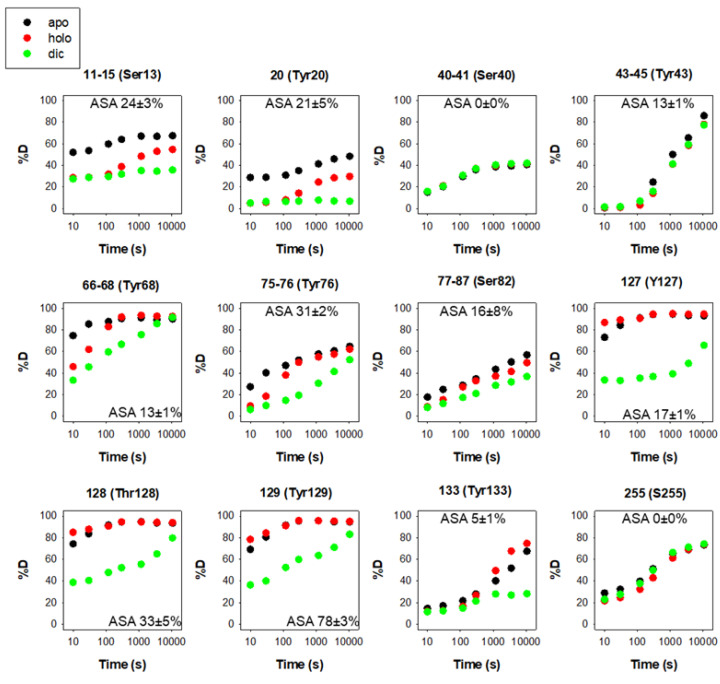
Structural stability of the phosphorylation sites in hNQO1 in different ligation states (apo, no ligand bound; holo, saturated with FAD; dic, saturated with FAD and dicoumarol). Data show the time-dependence (*x*-axis) of the peptide backbone hydrogen–deuterium exchange (%D, *y*-axis) as determined by mass spectrometry (reproduced from [9]). The solvent accessible surface (ASA, %) is indicated in parentheses and calculated using GetArea and the PDB 2F1O [33] as the average ± s.d. from eight monomers.

There are 12 reported phosphorylation sites in hNQO1 (Figure 2). It is interesting to note that most of these sites are not highly solvent-exposed, but are located in regions with moderate to low structural stability, which is highly dependent on ligand binding, based on a recent HDX study (Figure 3) [9]. These analyses suggest that phosphorylation might depend strongly on ligand binding and consequent changes in protein dynamics, or might occur cotranslationally. 

The functional consequences of phosphorylation at sites S40, S82 and T128 have been addressed recently by the use of phosphomimetic mutations [10,13,35]. The outcomes of these studies have been very revealing, because the functional effects are widely different depending on the site modified. Phosphorylation at S82 causes the strongest effects, with a remarkable decrease in FAD binding affinity, and its local destabilization extends across the hNQO1 structure, affecting the catalytic cycle and intracellular stability [10,13,35]. The effect of phosphorylation at S82 synergizes with that of the polymorphism P187S, leading to an almost 1000-fold decrease in FAD binding affinity [35]. A network of recently diverged electrostatic interactions in the vicinity of S82 has been shown to explain the different response of human and rat NQO1 to phosphorylation at S82. This is due to a single mutation (R80H) that occurred about 20 million years ago during primate speciation [11,35,36]. Phosphorylation at sites S40 and T128 has milder functional effects, affecting enzyme kinetics and structure much more weakly [10]. Importantly, phosphorylation of T128 by AKT is also associated with enhanced ubiquitination by Parkin and subsequent degradation of hNQO1, supporting the notion that phosphorylation, ubiquitination and intracellular stability of hNQO1 might be intertwined [37]. We are currently characterizing phosphomimetic mutations at Y127 and Y129, located in the hNQO1 active site, and our preliminary results support that phosphorylation at Y127 may perturb binding of FAD to the active site of hNQO1, whereas Y129 could be implicated in the conformational heterogeneity likely associated with functional negative cooperativity in the holo-protein (Pacheco-García JL, Martín-García JM, Medina M and Pey AL, unpublished observations).

### 3.2. Ubiquitination

**Figure 4:**
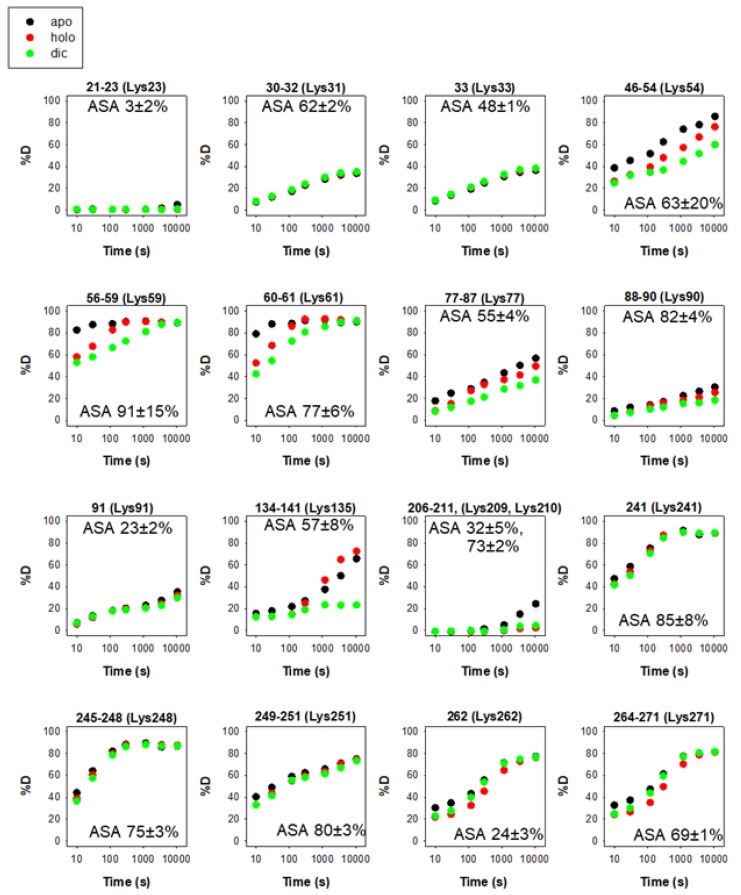
Structural stability of the ubiquitination sites in hNQO1 in different ligation states (apo, no ligand bound; holo, saturated with FAD; dic, saturated with FAD and dicoumarol). Additional details on data representation (reproduced from [9]) can be found in the legend of Figure 2. The solvent accessible surface (ASA, %) is indicated in parentheses and calculated using GetArea and the PDB 2F1O [33] as the average ± s.d. from eight monomers.

The intracellular stability of WT, and particularly of the polymorphic P187S hNQO1, is tightly associated to their C-terminal dynamics through ubiquitin-dependent proteasomal degradation of its CTD as an initiation site [15,31,32,38]. While this accelerated degradation of P187S might be due to enhanced dynamics of the CTD of the polymorphic variant P187S [13,31,39], it seems that the apo-state (ligand-free) of hNQO1 is particularly suitable for proteasomal degradation upon ubiquitin tagging even in the WT variant [38]. Thus, the sites K241, K248, K251, K262 and K271 are likely responsible for most ubiquitin-dependent degradation of hNQO1 in cells [38]. A vast majority of the ubiquitination sites are solvent-exposed and found in protein segments with moderate to low structural stability (Figure 4), and thus, are readily accessible for ubiquitin tagging upon interaction with a suitable ubiquitin-ligase (such as CHIP, [24]).

### 3.3. Acetylation

**Figure 5:**
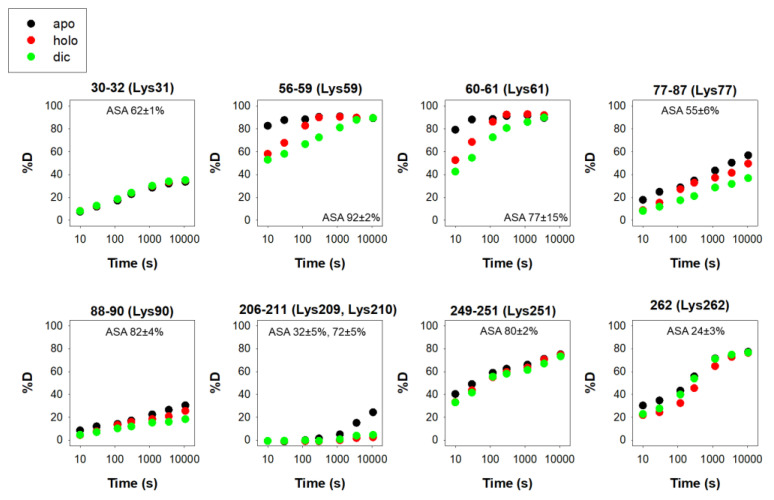
Structural stability of the acetylation sites in hNQO1 in different ligation states (apo, no ligand bound; holo, saturated with FAD; dic, saturated with FAD and dicoumarol). Additional details on data representation (reproduced from [9]) can be found in the legend of Figure 2 [9]. The solvent accessible surface area (ASA, %) is indicated in parenthesis and calculated using GetArea and the PDB 2F1O [33] as the average ± s.d. from eight monomers.

Until recently, acetylation of hNQO1 had been characterized only by high-throughput means, identifying nine sites with generally very high solvent exposure (logically, since Lys residues are often found on the protein surface) and typically in regions with moderate-to-low stability (Figure 5). However, Siegel and coworkers have recently described that acetylation of K33, K59, K61, K77, K90, K209, K251, K262 and K271 readily occurs upon in vitro exposure of recombinant hNQO1 to acetic anhydride or S-acetylglutathione [40]. It is interesting that these authors found helix 7 (residues 65–78) as one of the main targets for hNQO1 acetylation, since this region is next to the phosphosite S82 and the evolutionarily divergent site H80. Since all three phenomena (acetylation, phosphorylation and the variations R80 and H80) involve alterations in the electrostatic network important for FAD binding [11,35,36], I speculate that there could be some crosstalk between them in the modulation of NQO1 functionality in different mammalian species.

In vitro acetylation of hNQO1 led to a 37% decrease in steady-state catalytic function [40]. Importantly, acetylation was highly susceptible to functional ligand binding (NADH mediated reduction of FAD largely prevented acetylation), and deacetylation at K262 and K271 was quickly catalyzed by different sirtuins in vitro [40]. This phenomenon is likely associated with the NADH-dependent localization of hNQO1 around microtubules [3], and also highlights the plasticity of hNQO1 functionality in different ligation states and different subcellular locations [2,3,13]. 

Studies from our laboratory using mimetic mutations (at K31 and K209) have shown that the effect of acetylation on hNQO1 activity may be mainly ascribed to the K31 site (see the mutant K31Q in Table 1). This mutant also showed a mild decrease in thermal stability, both as holo- and apo-protein (Table 2). The role of electrostatic interactions in the effects of acetylating K31 was also supported by the greater effects found for the charge-reversal K31E mutant (Table 1 and Table 2).

Interestingly, these studies also highlight the versatility of certain K sites (such as K31, K59, K61, K209, K210, K251, K262 and K271) that can be modified differently (ubiquitination vs. acetylation) depending on the cellular conditions [38,40]. 

## 4. Naturally-Occurring and Artificial Mutations in NQO1: Allosteric Communication of Mutational Effects in a Multifunctional Stress Protein

Historically, two common polymorphisms in hNQO1 (namely rs1800566/c.C609T/p.P187S and rs1131341/c.C465T/p.R139W) have attracted most of the attention due to their frequency and association with cancer susceptibility [6,13,15,28,31,32,36,39,41,42,43,44]. These detailed studies have shown the long-range communication of mutational effects in different ligation states, a behavior which has been systematically corroborated by the recent characterization of naturally-occurring and artificial mutations [12,30,45]. We describe the insights provided by these mutational studies and their relationship with allosteric effects on hNQO1 in this section. 

### 4.1. Polymorphic Variants

**Figure 6:**
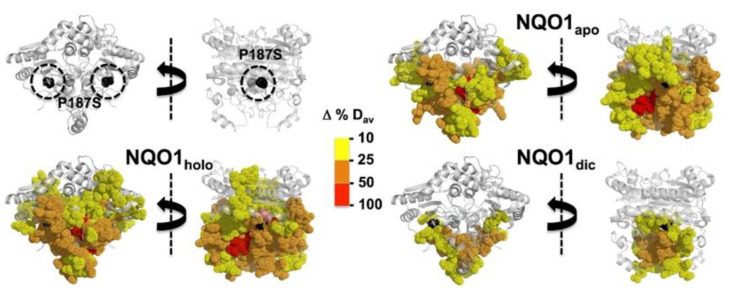
Changes in structural stability of hNQO1 due to the polymorphism P187S in different functional states (NQO1_apo_, no ligand bound; NQO1_holo_, saturated with FAD; NQO1_dic_, saturated with FAD and dicoumarol). Destabilization (as Δ%D_av_, compared to the WT in the same ligation state) is represented as indicated by the color scale. Data are taken from [13].

The c.C609T/p.P187S polymorphism occurs in the human population with a frequency of ~0.25 based on the gnomAD database, and almost reaches a frequency of 0.5 in the East Asian population, with about 5% of human population being homozygotes (https://gnomad.broadinstitute.org/variant/16-69745145-G-A?dataset=gnomad_r2_1 accessed on 15 December 2022). Different studies have supported its association, particularly in homozygosis, with cancer development [8,14,46]. Early studies showed nearly null activity-protein levels in homozygosis and substantially reduced activity in heterozygosis in cells and cancer samples [42,47]. Pro187 is buried in the protein structure, and its change to Ser causes a strong local structural destabilization that propagates through the entire NQO1 structure differently depending on the ligation state (Figure 6). Several functional features of hNQO1 are affected by P187S. First, the affinity for FAD is reduced by 10–40-fold by this polymorphism, depending on the experimental conditions and binding model employed. This is due to a long-range propagation of the variant effect to the FAD binding site in both the apo- and holo-states [5,28,35,36,44,48] (Figure 5). In addition, the effects of P187S also propagate to the CTD, affecting both catalysis and intracellular stability. Binding of the NADH cofactor or the inhibitor Dic are severely reduced, and thermodynamic analyses support that this is due to partial unfolding of the CTD in this variant [13,32,33]. Destabilization of the CTD also causes accelerated degradation of the NQO1 protein through enhanced ubiquitination of the CTD [15,31,32,36,38], and, consequently, CTD thermodynamic stabilization upon Dic binding leads to its intracellular stabilization in cell cultures [13,49]. Gradual structural perturbation at the P187 lead to different effects at different functional sites, supporting that the propagation of the local structural–energetic effects of P187S are anisotropic and long-range [13,32,48]. The large structural destabilization caused by P187S in the apo- and holo-states might also explained its enhanced coaggregation with other proteins in cell cultures under normal and low riboflavin supplies [50]. 

The c.C465T/p.R139W polymorphism is much less frequent, with an overall frequency of ~0.03 that increases up to ~0.06 in South Asian population, and is rarely found in homozygosis (https://gnomad.broadinstitute.org/variant/16-69748869-G-A?dataset=gnomad_r2_1 accessed on 15 December 2022). This polymorphism is associated with decreased intracellular activity and increased cancer risk [41,51,52]. This polymorphism causes dual effects, leading to a missense variation in the hNQO1 sequence (R139W), and in parallel, it massively causes skipping of exon 4 (residues 102–139), which destroys the binding sites of FAD and the substrate, yielding an unstable protein form [41]. Since the effects of the missense variation R139W are quite mild at the protein level [28,44,49], the main loss-of-function mechanism seems to arise from aberrant splicing. 

### 4.2. COSMIC Variants

**Figure 7:**
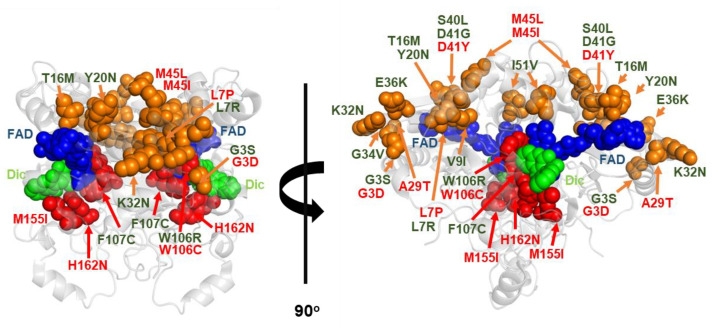
Structural location of COSMIC and gnomAD mutations, characterized experimentally. Variants labeled in red are those from COSMIC and in green are those found in gnomAD. Active site mutants are shown as red spheres. Reproduced from [30].

By 11 December 2022, there were 107 different mutations in hNQO1 compiled in the **C**atalogue **O**f **S**o**M**at**I**c **C**ancer Cell lines catalogue (**COSMIC** database; https://cancer.sanger.ac.uk/cosmic/gene/analysis?ln=NQO1_ENST00000561500 accessed on 11 December 2022), of which 73 were missense mutations. The effects of 10 of these mutations (G3D, L7P, A29T, D41Y, M45L, M45I, W106C, M155I, H162N and K240Q) have been recently characterized in some detail in vitro [30,31,45,48,53] (Figure 7 and Table 3). This set of mutations mostly target the NTD (Figure 7), and in some cases, affect active site residues (W106C, M155I and H162N). 

We must note that the presence of a mutation in a cancer cell line (i.e., COSMIC) does not unambiguously imply that it is a cancer driver mutation (as discussed in [30]). Nevertheless, we found alterations in several NQO1 functional features due to these COSMIC mutations. All mutations, except G3D and K240Q, showed moderate to severe alterations in their foldability (formation of dimers or enhanced aggregation) or in the thermal stability of the folded dimer [30,32,48,53] (Table 3). The mutants M155I and H162N also caused severe defects in FAD binding and catalytic efficiency, whereas the effects of the W106C and K240Q on these functional features were much milder [30,32,48,53] (Figure 7 and Table 3). High resolution stability analyses have shown that impaired FAD binding affinity and catalysis in the M155I and H162N mutants likely stem from extensive and specific destabilization of the structure of the holo-hNQO1 [53].

### 4.3. gnomAD Variants

**Figure 8:**
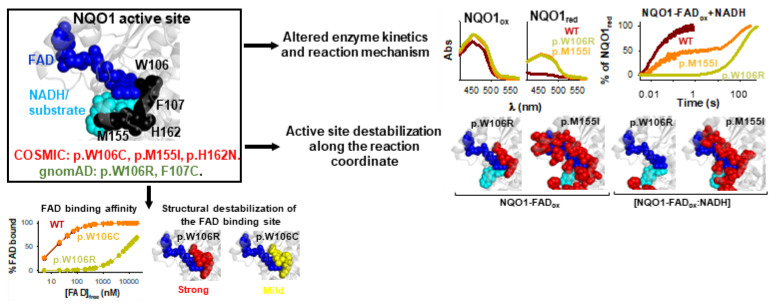
Molecular characterization of active site mutants from COSMIC and gnomAD databases in hNQO1. Experimental analysis include enzyme kinetics and analysis of the reaction mechanism by stopped-flow absorption spectroscopy and effects on the structural stability by HDX- and FAD-binding affinities. Adapted from [53].

By 11 December 2022, there were over 150 variations described in the NQO1 gene in non-disease associated, human population, large-scale sequencing initiatives (gnomAD database; https://gnomad.broadinstitute.org/gene/ENSG00000181019?dataset=gnomad_r2_1 accessed on 11 December 2022), of which 106 are missense variants. 13 of these have been characterized experimentally (G3S, L7R, V9I, T16M, Y20N, K32N, G34V, E36K, S40L, D41G, I51V, W106R, F107C; Figure 7) [30,45]. The study of variants present in the human population is relevant because the presence of germline mutations may cause predisposition to additional somatic mutations and facilitate cancer development [30] (Table 3). Importantly, several of these rare mutations moderately to largely destabilized the protein or reduced NQO1 foldability (L7R, T16M, Y20N, G34V, S40L, D41G, I51V, W106R and F107C). In addition, several mutations affected FAD binding with moderate (~3-fold lower affinity, Y20N; ~10-fold, T16M and I51V) to large effects (~500-fold lower affinity, W106R) [30,53]. The kinetic characterization of the W106R and F107C mutants, two highly non-conservative mutations at the active site of NQO1, provided counterintuitive results; the former had catastrophic consequences on enzyme activity, while the latter had very mild effects [53]. Interestingly, the strongest effect on local stability due to W106R occurred in the NQO1_dic_ state, possibly reflecting heavy effects on catalytic intermediate states [53]. One of the most revealing outcomes of these studies was the finding that variations found in disease-focused databases (i.e., COSMIC) can be as deleterious for NQO1 function as those found in the normal population. (Figure 8).

### 4.4. Artificial Variants Aimed at Evaluating the Propagation of Stability Effects and the Structural Basis of Allosterism

Two sets of artificial mutants have recently been generated and characterized in vitro [12,32,48]. The first one was intended to determine whether propagation of mutational effects at site P187 and K240 depended on the magnitude of the local stability effects and single vs. multiple nucleotide changes [32,48]. The latter was intended to perturb the hydrophobic core of hNQO1 at different locations and to different extents [12]. These studies have highlighted the significant plasticity of hNQO1 towards mutational effects in different ligation states. 

In the first work, we observed that unnatural variants at the P187 and K240 sites may lead to even more deleterious effects on several hNQO1 functional features than natural variants (i.e., P187S and K240Q), in some cases through a single nucleotide change. Using functional assays, this study provided the first large-scale evidence that propagation of local mutational effects in hNQO1 may occur to distant sites (up to 20 Å) in a general manner, not restricted to the P187S polymorphism [48]. 

**Figure 9:**
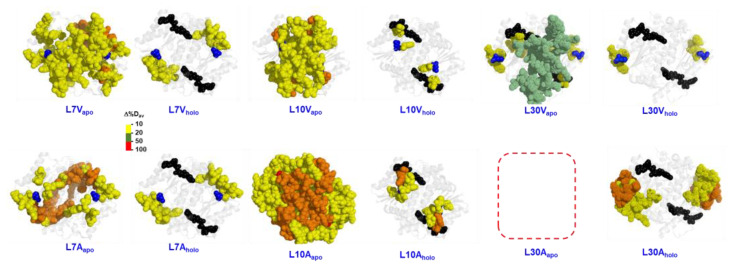
Cavity-making artificial mutations mostly target the structural stability of apo-hNQO1. HDX data are shown as Δ%D_av_, as described in [12]. Residues in blue correspond to those that are mutated. Data were displayed using the structure with PDB code 2F1O [33]. Data for L30A_apo_ were not acquired due to the instability of this sample.

The second work was intended to rationalize these allosteric effects at different sites at the NTD by truncating fully buried hydrophobic residues (L7, L10 and L30) to smaller residues (to V or A) and even residues truncating+increasing conformational entropy (to G) [12] (Figure 9). All three mutations to Gly caused a remarkable decrease in protein foldability, which was extended to any mutation at L30, highlighting the site-dependence of the propagation of mutational effects. Perturbation of the L10 site (mutant L10A) also led to a remarkable ~20-fold decrease in FAD binding affinity, likely caused by the propagation of this structural perturbation by ~10 Å to the FAD binding site [12]. Generally, all of these cavity-making mutations had insignificant catalytic effects, indicating that most of the effects affected protein foldability and/or FAD binding [12]. Most stability effects were found only in the hNQO1-apo-state. Some mutants (L7V and L7A, L10V and L10A), did not prevent formation of dimers, but notably destabilized them [12] (Figure 9). 

These studies elegantly showed that energetic perturbations due to missense variations in hNQO1 may target several functions in different manners through specific effects on different functional (ligation) states. 

## 5. Outlook and Future Perspectives

Two critical aspects for personalized medicine and pharmacogenomics are the roles of PTMs and the emerging genetic variability among individuals, which can determine their susceptibility to disease and response to therapeutics. Although recent advances have been achieved, in silico prediction methods are still underperforming in experimental evaluation, particularly for multifunctional proteins such as hNQO1 [30,53]. Artificial intelligence based approaches are revolutionizing the research in protein chemistry, biochemistry and structural biology, but these are still far from providing good predictors of genotype–phenotype relationships, particularly in multifunctional proteins [54,55]. A multidisciplinary and holistic experimental approach may help in training AI tools to improve the predictions of these relationships on a large scale [56]. In this review, I have briefly updated the information on these relationships using experimental approaches, clearly showing how genetic diversity and interindividual differences in PTMs might be critical for developing oxidative stress-related diseases associated with changes in hNQO1 functionality. Although, in a recent study, we were able to predict 2/3 of the experimental phenotypes, this is still far from being sufficient for appropriate in silico genotype–phenotype correlations [30]. We aim to continue working on the genotype–phenotype correlations in protein multifunctionality, as well as genetic diversity and its modulation by PTMs, using hNQO1 as an excellent model of an antioxidant and multifunctional disease-associated protein. 

## Figures and Tables

**Table 1:** Steady-state enzyme kinetic parameters for the reduction in DCPIP by hNQO1 WT and mutants at K31 and K209. Proteins were expressed and purified according to [13]. Activity was measured according to [31] using 20 µM DCPIP and 0–2 mM NADH at 25 °C. Data were fitted to the Michaelis–Menten equation.

NQO1 Variant	k_cat_ (s^−1^)	K_M (NADH)_ (mM)	k_cat_/K_M_ (s^−1^·mM^−1^)
WT	50 ± 4	0.54 ± 0.10	91 ± 18
K31Q	36 ± 4	0.44 ± 0.10	82 ± 20
K31E	32 ± 5	0.38 ± 0.08	86 ± 19
K209Q	43 ± 6	0.29 ± 0.10	147 ± 50
K209E	42 ± 5	0.30 ± 0.08	142 ± 40

**Table 2:** Thermal stability of hNQO1 variants at K31 and K209. Thermal denaturation was carried out by intrinsic fluorescence emission spectroscopy, as described in [36].

NQO1 Variant	Ligation State ^1^	T_m_ (°C) ^2^
WT	Holo	55.8 ± 0.5
	Apo	52.0 ± 0.6
K31Q	Holo	54.5 ± 0.1
	Apo	50.5 ± 0.7
K31E	Holo	53.3 ± 0.1
	Apo	49.6 ± 0.6
K209Q	Holo	55.4 ± 0.1
	Apo	51.9 ± 0.3
K209E	Holo	53.5 ± 0.1
	Apo	50.8 ± 0.6

^1^ Apo indicates samples in which FAD has been stripped. Holo indicates proteins that were purified in the presence of 20 µM FAD. Protein concentration was 1 µM. ^2^ Average ± s.d. from four replicates.

**Table 3:** Experimental characterization of the effects of natural variants on NQO1. Mutations were retrieved from COSMIC or gnomAD databases. Three different parameters are reported and normalized using the WT protein: Soluble protein levels upon expression in *E. coli* at 37 °C, change in denaturation temperature (ΔT_m_) and change in the dissociation constant for FAD (as the ratio of mutant/WT constants). N.Det. indicates not determined. Original and un-normalized data can be found in [30,45,48].

NQO1 Variant	Soluble Protein Levels (vs. WT)	ΔT_m_ (vs. WT) (°C)	K_d (FAD)_ (WT-Fold)
WT	1.0 ± 0.1	0.0 ± 0.6	1.0 ± 0.2
G3S	2.0 ± 0.3	−0.4 ± 0.8	1.4 ± 0.3
G3D	3.8 ± 1.1	−1.5 ± 0.7	0.9 ± 0.4
L7P	0.5 ± 0.1	N.Det.	N.Det.
L7R	>>0.1	N.Det.	N.Det.
V9I	0.8 ± 0.6	−1.7 ± 0.6	1.4 ± 0.3
T16M	0.4 ± 0.2	−4.3 ± 0.7	10.8 ± 0.2
Y20N	0.6 ± 0.1	−5.1 ± 0.6	2.9 ± 0.5
A29T	1.3 ± 0.3	0.1 ± 0.7	5.0 ± 0.4
K32N	0.8 ± 0.2	−0.2 ± 0.6	0.8 ± 0.4
G34V	>>0.1	N.Det.	N.Det.
E36K	0.9 ± 0.2	0.0 ± 0.8	0.8 ± 0.5
S40L	>>0.1	N.Det.	N.Det.
D41G	>>0.1	−7.9 ± 0.5	N.Det.
D41Y	>>0.1	−9.7 ± 0.6	N.Det.
M45L	0.4 ± 0.5	−3.7 ± 0.5	0.5 ± 1.0
M45I	0.2 ± 0.4	−3.4 ± 0.6	0.6 ± 0.8
I51V	0.4 ± 0.3	−5.0 ± 0.5	8.9 ± 0.3
W106R	>0.1	−6.4 ± 0.7	~ 500
W106C	0.2 ± 0.5	−2.5 ± 0.7	0.9 ± 0.4
F107C	0.1 ± 0.7	0.6 ± 0.7	~ 0.2
M155I	>0.1	−0.9 ± 0.6	45 ± 1
H162N	0.3 ± 0.9	−0.7 ± 1.0	27 ± 1
K240Q	1.3 ± 0.3	0.5 ± 0.6	2.8 ± 0.6

## Data Availability

All data not published will be shared with the readership upon adequate request.
